# Case report and literature review: double jeopardy – *Exophiala dermatitidis* and *Mycobacterium canariasense* central line-associated bloodstream infection in a patient

**DOI:** 10.1099/acmi.0.000347

**Published:** 2022-04-19

**Authors:** Afrinash Ahamad, Bushra Tehreem, Maaz Farooqi, Bennadette Maramara

**Affiliations:** ^1^​ Clinical Laboratory Sciences Program, School of Health Professions, Stony Brook University, Stony Brook, NY, USA; ^2^​ Department of Neuroscience and Behavior, Stony Brook University, Stony Brook, NY, USA; ^3^​ Neonatal-Perinatal Medicine, SUNY Downstate Medical Center, Brooklyn, NY, USA; ^4^​ Medical Informatics, SUNY Downstate Health Sciences University, Brooklyn, NY, USA; ^5^​ Divison of Infectious Disease, Stony Brook Medical Center, Stony Brook, NY, USA

**Keywords:** irritable bowel syndrome, *Exophiala dermatitidis*, blood culture, catheter-associated infection (CLABSI), *Mycobacterium canariasense*, MALDI-TOF- MS

## Abstract

Central line-associated bloodstream infection (CLABSI) is the most common nosocomial-acquired infection, affecting 38 000 patients in the USA annually. Approximately 8–10 % of inserted catheters lead to bloodstream infections, and ~25–30 % of infections are associated with mortality. Although proper line maintenance is essential to prevent infection, it is quite a challenge to avoid infection in patients with a long-term catheter. We present a case of a female in her 40s with a previous history of irritable bowel syndrome (IBS) who has had a central line for total parenteral nutrition for the past 2 years. The patient recently visited the emergency room with fever and generalized fatigue. Blood cultures sent to microbiology were positive for black mould, *Exophiala dermatitidis*. However, after a few days, microbiology reported an additional micro-organism, *Mycobacterium canariasense *, a pathogen rarely associated with bacteraemia. The patient was administered voriconazole and moxifloxacin for black mould and mycobacterium infection, respectively. We present an unusual case of rare opportunistic organisms causing bacteraemia and fungaemia in a patient with a long-term catheter. CLABSI remains a serious challenge for clinical facilities. Implementation and monitoring of effective strategies can prevent catheter-related bloodstream infections in patients with long-term catheters and can reduce the morbidity and mortality associated with CLABSI.

## Background

Central line-associated bloodstream infection (CLABSI) is a primary bloodstream infection with no apparent contribution from another body site [1]. Annually 38 000 cases of CLABSI are reported in the USA [2]. Before the coronavirus disease 2019 (COVID-19) pandemic, a significant reduction was reported in CLABSI events [3]; however, based on a study reported by McMullen *et al*. in 2020, a significant increase in CLABSI has been documented in hospitalized COVID-19 patients with complications [4]. Most CLABSI cases are preventable with proper aseptic techniques, surveillance and management [5]. In diagnostic laboratories, culturing the catheter tip and blood culture (aerobic and anaerobic) are the primary diagnostic methods to identify laboratory-confirmed bloodstream infection (LCBI) [6]. LCBI is used to standardize the surveillance of CLABSI and must meet one of the following criteria: (1) the infection present must be related to the catheter and no other site should be contributory to the infection; (2) the patient must have a clinical presentation (fever, chills); and (3) the laboratory diagnostic results must not be related to infection of any other body site [7].

We present an unusual coinfection, bacteraemia and fungaemia, resulting from *Exophiala dermatitidis* and *Mycobacterium canariasense* in a patient with a venous catheter. We herein, summarize the prevention measures essential to avoid the morbidity and mortality associated with CLABSI and provide a literature review for both organisms.

## Case report

**Fig. 1.**
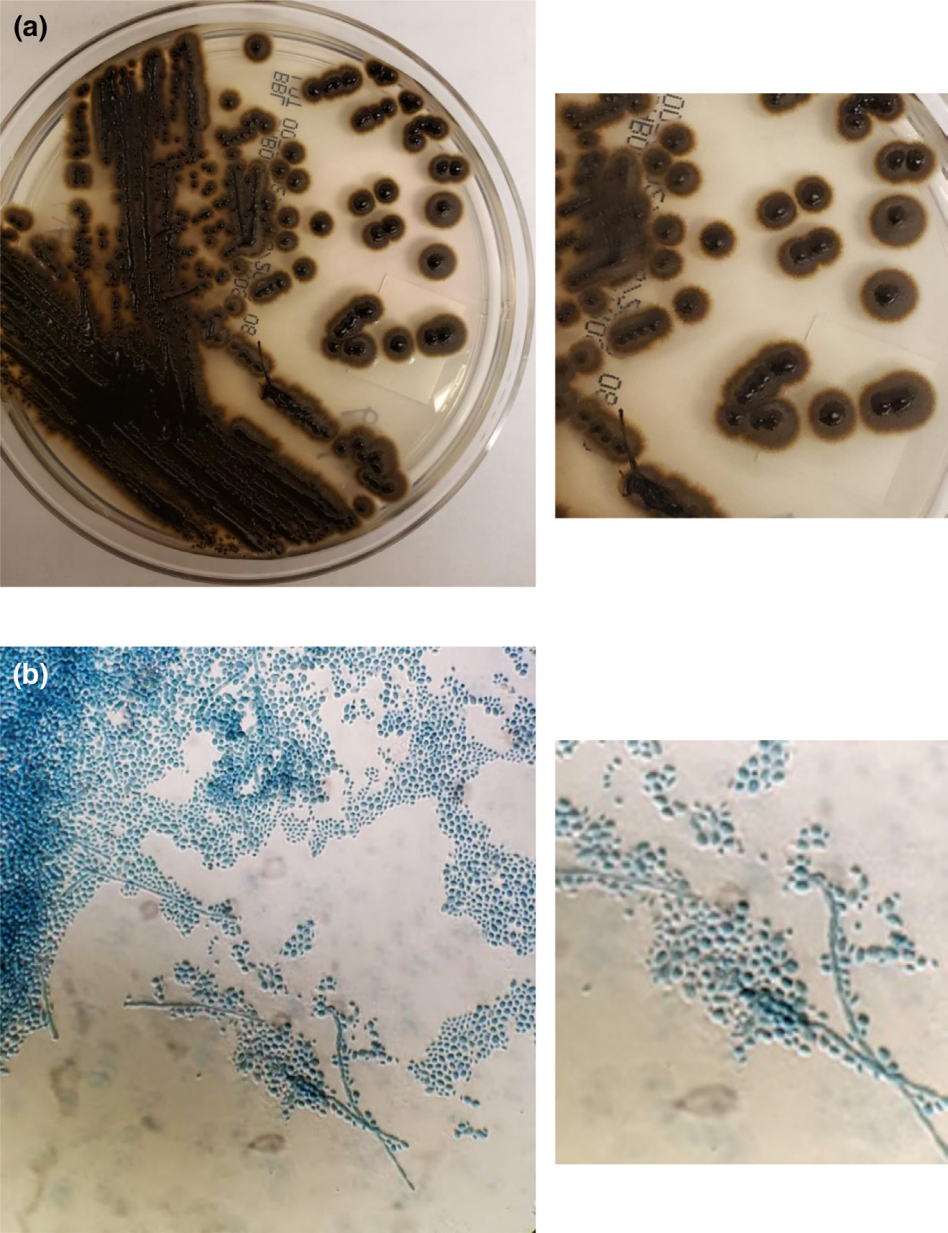
(a) Dry and rough black or olive black colonies upon maturation – note the dry edges as the colonies mature. (b) Lactophenol blue stain shows *Exophiala dermatitidis* conidia.

The patient was a female in her 40s with a history of malabsorption disorder due to unspecified irritable bowel syndrome (IBS), necessitating use of total parenteral nutrition (TPN) for the past 15 years, and who had a central line catheter (PICC, Hickman) placed 2 years ago. Recently, she presented to the emergency room (ER) complaining about generalized fatigue and fever. Three blood cultures sets (two peripheral and one central line) drawn in the ER were sent to the bacteriology laboratory for testing. The patient had no chills, nausea, vomiting, or vision changes. She denied any change in appetite. The patient was alert, oriented and atraumatic. Her chest sounded unremarkable. Her gastrointestinal (GI) physician had performed an extensive workup for her malabsorption disorder. Although she can consume food and liquid orally in moderate amounts, TPN is required six times per week for dietary supplementation. The patient has a history of chronic anticoagulation. She had a previous line infection due to *Candida* species and had associated endophthalmitis. The patient’s initial report showed chloride 109 (98–107 mmol l^−1^) and CO_2_ 21 (22–29 mmol l^−1^). The hepatic panel showed total protein 6.3 (6.37–8.5 g dl^−1^), albumin 3.2 (3.5–5.2 g dl^−1^), aspartate aminotransferase (AST) 67 (5–34 U l^−1^), alkaline phosphatase 188 (40–150 U l^−1^) and alanine aminotransferase (ALT) 59 (0–37 U l^−1^). The patient complete blood count (CBC) result was unremarkable. Urinalysis results showed moderate blood, small amounts of protein, crystals and coarse granular and fine granular casts. Procalcitonin was 0.10 ng ml^−1^ (>0.5 possible systemic infection). All blood culture sets (aerobic and anaerobic) drawn at the admission yielded yeast after 48 h of incubation in the BacT/ALERT 3D Microbial Identification System. A nasopharyngeal swab submitted for COVID-19 PCR performed on the Cepheid Xpert Xpress was negative. A catheter tip sent for culture also grew >15 colony-forming units (c.f.u.) of yeast, similar to the colonies observed from blood culture. The patient was treated empirically with micafungin. Rapid diagnosis performed on the T2 Magnetic Resonance (T2MR) system (T2Biosystems, Inc., Wilmington, MA, USA) to rule out candidiasis was negative for C*andida albicans*, *Candida glabrata*, *Candida tropicalis*, *Candida parapsilosis* and *Candida krusei*. After 48 h of incubation of blood and CHROM agar (Becton Dickinson, Sparks, MD, USA) from both catheter tip and blood culture, the initial creamy colonies transitioned to be dry and black olive in colour with maturation (Fig. 1a), and non-*Candida* yeast or mould was suspected. Direct microscopic examination of the organism showed oval budding yeasts cells, and lactophenol blue staining of the mature colonies revealed pigmented septate branched hyphae with annelidic conidiogenesis, and ellipsoidal conidia of different sizes with a thin wall, forming aggregates (Fig. 1b) that were consistent with *Exophiala* species.

**Fig. 2.**
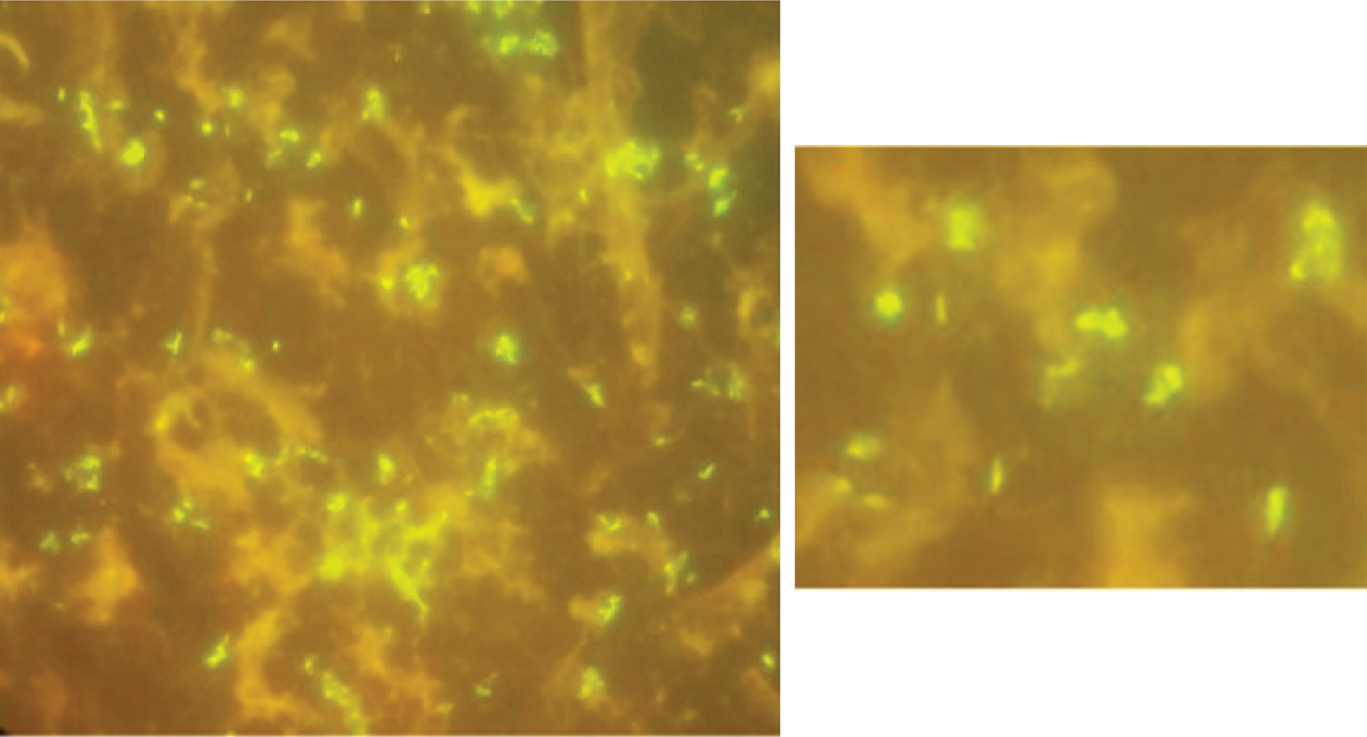
The acid-fast *Mycobacterium canariasense* appearing yellow or green when stained with auramine–rhodamine stain.

The results from VITEK MS matrix-assisted laser desorption/ionization time-of-flight mass spectrometry (MALDI-TOF MS) (bioMérieux, USA) along with additional identification were consistent with black mould, *E. dermatitidis*. The patient was administered voriconazole 150 mg intravenously every 12 h for 14 days after antifungal susceptibility testing was performed. A repeat comprehensive metabolic panel (CMP) result after a few days showed AST 48 U l^−1^ and ALT 44 U l^−1^ and a decrease in platelets to 139 (150–400 10×3 μl^−1^). The patient’s one peripheral blood culture (aerobic bottle) was positive for Gram-positive rods, which failed to grow on media. While plates were reincubated for a longer duration, the specimen was sent to the mycobacteriology laboratory for further investigation. The initial auramine–rhodamine stain performed from the blood culture was positive for the acid-fast organism (Fig. 2), but the specimen inoculated into Lowenstein–Jensen medium and a protein-rich medium, Middlebrook medium 7H10 (Becton Dickinson, Sparks, MD, USA), incubated at 37 °C showed no growth, and specimens were sent to the reference laboratory for identification.

Diagnostics identified the second isolate as *M. canariasense* by rpoB gene sequencing. The catheter was removed and based on the susceptibility testing performed by the Clinical and Laboratory Standards Institution (CLSI) microdilution method for rapidly growing mycobacterium (RGM), the organism was sensitive to all tested drugs, including amakacin (<=8), ciprofloxacin (=1), clarithromycin (<=0.25), moxifloxacin (<=0.5), doxycycline (<=1), imipenem (<=2) and linezolid (<=1), and the patient was treated with 400 mg moxifloxacin daily for 10 days. After treatment, all the subsequent blood cultures were negative following 5 days of incubation.

## Discussion

Central line-associated bloodstream infection (CLABSI) is one of the leading causes of preventable infections in healthcare facilities. Annually, approximately 38000 CLABSI cases are reported in the USA, which cost 2.3 billion. CLABSIs result in an increased length of hospital stay and cause a substantial nationwide pecuniary burden, with an additional $45 000 cost per case [3]. CLABSI occurs via four recognized routes: (1) the most common mechanism, migration of skin endogenous flora at the entry site of the catheter and colonization at the catheter tip; (2) non-adherence to sterility, i.e. contamination of the catheter and hubs with contaminated fluid and hands; (3) haematogenous seeding from a distant or another focus of infection; and (4) contamination of the intravenous infusate, which is rare [5, 8]. In addition, pathogenic determinants, such as the material used for device composition, the host extracellular matrix components, such as fibrin and fibronectin, which form a sheath around the catheter, and the extracellular polymeric substance (EPS) produced by the adherent organisms, cause infection, either locally or at a distant site via circulating toxins [9, 10]. In patients with CLABSI, alternative sources of infection must be evaluated carefully, as CLABSI management may require catheter removal in a patient with limited vascular access, which poses a challenge. In an attempt to eradicate the infection, catheter salvage therapy (CST) can be used, in which the infected lumen is filled with a supraphysiological concentration of antibiotic to which the organism is susceptible, allowing it to dwell for hours to days to create an ‘antibiotic lock’. CST is used in combination with systemic therapy for 10–14 days [11–13]. To prevent CLABSI, proper central line insertion practices (CLIP), such as hand hygiene before catheter insertion or manipulation, maximal barrier precaution for catheter insertion and removal of nonessential lines, chlorhexidine skin antisepsis for site preparation (unless allergic), change of dressing, optimal catheter site selection and daily review of line adherence, is essential [3, 14]. Coagulase-negative *Staphylococcus* (16 %), *Staphylococcus aureus* (13 %) *Klebsiella pneumoniae* (8 %), *Enterococcus faecalis* and *Enterococcus faecium* (8 and 7 %), and *Candida albicans* (6 %) are the commonly reported CLABSI pathogens [15]. Based on our observations, the fungal agent, *E. dermatitidis*, and acid-fast *Mycobacterium canariasense* are separately and rarely reported to be associated with CLABSI [16–18]. This is an unusual case of the co-occurrence of these infectious agents in a patient and the central catheter is considered to be the portal of entry.

*E. dermatitidis*, an opportunistic, ubiquitously present dematiaceous fungus [19], is an aetiological agent of three types of infections: superficial, subcutaneous and systemic. The disease spectrum varies from localized cutaneous infection [20] to disseminated disease, and is referred to as phaeohyphomycosis, which is commonly reported in immunocompromised patients. ‘Phaeo’' in Greek means ‘dark’ and refers to the coloration resulting from the presence of melanin 1,8-dihydroxynaphthalene (a key intermediate required in the synthesis of melanin) in the cell wall of the organism [21, 22]. Melanization is one of the primary pathogenic mechanisms that help the organism to evade the host immune system by blocking the effect of liberated free radicals and hydrolytic enzymes in the phagolysosomes of the neutrophils without influencing either phagocytosis or the oxidative burst of the neutrophils [23–25].Several other virulence attributes, such as adhesion to the surface, hydrophobicity of the cell wall, chitin synthase, and formation of meristems, biofilm and secondary metabolites (acidic and alkaline) are also contributory to the infection. Due to a broad array of virulence attributes, *E. dermatitidis* has become a serious concern in patients with underlying conditions [26]. In otherwise healthy immunocompetent individuals, *E. dermatitidis* has a proclivity to cause infection in the Asian population; the underlying reason for this preference remains elusive to date [27, 28]. The colonies of *Exophiala* spp. are moist and yeast-like in appearance, and with maturation exhibit dark green to olive black pigmentation [29]. Capsules and exopolysaccharides are species-specific characteristics and have phenotypic diagnostic values [27]. *E. dermatitidis* can be morphologically present either as yeast, which is linked to disseminated haematogenous infection, or hyphal growth, which usually results from localized infection [17], and the presence of sclerotic bodies due to *E. dermatitidis* is associated with chromoblastomycosis [30]. *E. dermatitidis* can grow at 24–45 °C, optimal growth is observed at 33 °C, and a temperature >45 °C is fungistatic. In diagnostic mycology, besides microscopy and culture media, molecular methods such as ribosomal DNA internal transcribed spacer (ITS) sequencing [31] or MALDI-TOF have been employed for the identification of *E. dermatitidis* [32–35]. In the case presented, a non-culture-based test was performed using T2 magnetic resonance (T2 MR) to rule out candidiasis. T2MR is a nanodiagnostic panel widely used in the diagnostic laboratory for the identification of *Candida* species in whole blood. The advantage of the diagnostic tool is a rapid turnaround time in the identification of *Candida*-associated bloodstream infections, but the test is limited to the identification of five major *Candida* species (*C. albicans, C. glabrata, C. tropicalis, C. parapsilosis*, *C. krusei*), and other rare fungal agents, such as *E. dermatitidis*, cannot be identified via this platform [36]. Although *E. dermatitidis* is associated with severe systemic infections and has a mortality rate of 80 %, rare to no specific guidelines for the treatment of bloodstream infection have been established [37]. In this case, micafungin, a semisynthetic echinocandin, was empirically administered based on the suspicion of candidiasis. Upon confirmation of *E. dermatitidis*, the antifungal therapy was changed to voriconazole, a second-generation triazole that acts as a cytochrome p450n14α-demethylase inhibitor and prevents the conversion of lanosterol to ergosterol, which is essential for fungal cell wall synthesis. The sensitivity of voriconazole varies with species and it is necessary to speciate the organism before prescribing the drug [38, 39].

*M. canariasense* comprises rapidly growing partially acid-fast rods, similar to other non-tuberculosis *Mycobacterium* (NTM) species [40]. *M. canariasense* is a relatively novel organism, and thus far few clinical cases have been reported within the USA. No particular environmental source has been identified with the spread of *M. canariasense* and no evidence of human-to-human transmission has been reported [41]. Based on its mycolic acid and fatty acid pattern, *M. canariasense* can be differentiated from other nosocomial infection-associated *Mycobacterium* species, such as *Mycobacterium chelonae *, *Mycobacterium fortuitum* and *Mycobacterium abscesses*. Further, phenotypic and biochemical characteristics play an essential role in the identification of *M. canariasense* [42–44]. Rapid methods of mycobacterium species identification involve the use of molecular techniques, and restriction fragment length polymorphism (PCR- RFLP) of heat shock protein 65 (hsp65) gene [45, 46]. Further, the sequences of conserved genes and DNA–DNA hybridization can be utilized to speciate the isolate [47]. In a diagnostic laboratory *M. canariasense* transitions from colourless to shiny yellow colour-producing colonies with maturation on Lowenstein–Jensen medium. Organism growth is usually observed on media after 2–3 days of incubation at 30–37 °C [36, 41, 42]. To date, no cases of recurrence of *M. canariasense* in pulmonary infection and catheter-associated infection have been reported. Based on our antimicrobial susceptibility testing and also that reported by others, *M. canariasense* is a very susceptible organism, and to date no particular resistance pattern has been reported [41, 48].

## Conclusion

We present a unique case of two opportunistic organisms, *Exophiala dermatitidis* and *Mycobacterium canariasense *, commonly present in water sources and rarely associated with bloodstream infection. CLABSI is a global concern, and numerous strategies have been implemented to prevent catheter-associated infection in patients with long-term catheters. Our case summarizes the safety practices and measures that help in preventing morbidity and mortality associated with catheter-associated infections.
